# Evaluation of the real-world safety of eptifibatide in the treatment of ARDS: results of a disproportionality analysis of FAERS data

**DOI:** 10.3389/fmed.2026.1767036

**Published:** 2026-02-26

**Authors:** Peng Zhang, Wenjing Wang, Ting Zhao, Guoping Wang, Fanyong Shang, Feng Ji

**Affiliations:** 1 Department of Pulmonary and Critical Care Medicine, Shandong Provincial Third Hospital, Jinan, China; 2 Department of Infectious Diseases, Shandong Second Provincial General Hospital, Jinan, China; 3 Department of Respiratory Medicine, Shandong Provincial Corps Hospital of the Chinese People's Armed Police Force, Jinan, China; 4 Department of Internal Medicine, Lijin County Hospital of Traditional Chinese Medicine, Dongying, China; 5 Department of General Surgery, Lijin County Hospital of Traditional Chinese Medicine, Dongying, China; 6 Department of Intensive Care Unit, Jinan Central Hospital East Campus, Jinan, China

**Keywords:** adverse reactions, ARDS, disproportionality analysis, eptifibatide, FAERS

## Abstract

**Background:**

Acute respiratory distress syndrome (ARDS) is a clinical syndrome with an extremely high mortality rate, and antiplatelet therapy is an important treatment approach. Eptifibatide, a glycoprotein IIb/IIIa receptor inhibitor (GPI), is primarily used to treat acute coronary syndrome (ACS) and non-venous thromboembolic pulmonary embolism, as well as for antiplatelet therapy in conditions such as ARDS and septic shock. With its increasing clinical application, understanding its safety profile in real-world settings is essential.

**Methods:**

This study evaluated the clinical safety of Eptifibatide by analyzing all adverse event (AEs) reports in the FDA Adverse Event Reporting System (FAERS) where Eptifibatide was listed as the primary suspected drug since 2004. Analytical methods included the Bayesian Confidence Propagation Neural Network (BCPNN), the UK Medicines and Healthcare Products Regulatory Agency (MHRA) comprehensive standard method, the Multi-item Gamma Poisson Shrinker (MGPS), the Proportional Reporting Ratio (PRR), and the Reporting Odds Ratio (ROR).

**Results:**

The study confirmed known adverse reactions of Eptifibatide, such as bleeding, intracranial hemorrhage, stroke, thrombocytopenia, allergic reactions, immunogenicity, and hypotension, which are also listed in the drug's prescribing information. Additionally, some previously unmentioned adverse reactions were identified, including acute myocardial infarction, cardiac arrest, nausea, hemorrhagic pancreatitis, chills, dyspnea, and vascular pseudoaneurysm. The study also highlighted the importance of early detection of adverse reactions to Eptifibatide.

**Conclusion:**

This research provides insights into both known and potential adverse reactions associated with Eptifibatide in real-world clinical use, offering additional safety information for clinicians when prescribing Eptifibatide for ARDS treatment.

## 1 Introduction

Acute respiratory distress syndrome (ARDS) is a clinical syndrome characterized by refractory hypoxemia, respiratory distress, and non-cardiogenic pulmonary edema, triggered by pulmonary factors such as aspiration and pneumonia, or by extrapulmonary factors like severe infection, shock, and major trauma (1). The pathophysiological of ARDS centers on severe inflammatory responses and coagulation abnormalities. This processes damage the alveolar epithelium and pulmonary vascular endothelium, ultimately leading to microthrombosis and increased alveolar-capillary permeability. The resultant pulmonary interstitial edema, and ventilation-perfusion mismatch are key contributors to refractory hypoxemia (2). ARDS is a major cause of respiratory failure in critically ill patients, with an incidence of up to 23% in mechanically ventilated patients and a mortality rate exceeding 30%, where pulmonary microthrombosis is a leading cause of death (3). Therefore, developing effective treatments for ARDS and improving patient outcomes remain key challenges in current research.

Activated platelets play a regulatory role in alveolar-capillary permeability, neutrophil infiltration, and endothelial barrier function, all of which influence the progression of ARDS (4). Recent studies have demonstrated that modulating coagulation and antiplatelet therapy may improve the condition and prognosis of ARDS patients. Looney et al. (5) found that aspirin reduces platelet aggregation in pulmonary vessels, mitigating transfusion-related acute lung injury. Hayes et al. (6) confirmed that acetylsalicylic acid alleviates pulmonary edema and improves survival by inhibiting platelet activation and reducing inflammatory responses. Hamid et al. (7) showed that aspirin decreases lipopolysaccharide-induced neutrophil accumulation in the lungs, attenuating pulmonary inflammation. Thus, suppressing platelet activation may improve outcomes in ARDS.

The potent antiplatelet effect of eptifibatide, a glycoprotein IIb/IIIa receptor inhibitor (GPI), is mediated by its competitive inhibition of fibrinogen and other adhesive ligand binding to this integrin receptor (8). It exhibits high selectivity and short duration of action (half-life ~2.5 h, with antiplatelet effects diminishing within 4 h after discontinuation), along with low immunogenicity and favorable biosafety (9, 10). Currently, eptifibatide is extensively used as an antiplatelet agent in patients with acute coronary syndrome (ACS), covering the full spectrum of STEMI, NSTEMI, and unstable angina, primarily to reduce ischemic complications. Beyond ACS, its therapeutic potential is being investigated in a range of other thromboinflammatory disorders, such as stroke, ARDS, and septic shock (11–13). It is also used as an adjunctive antiplatelet agent in percutaneous coronary intervention (PCI) to minimize perioperative thrombosis risk, often in combination with aspirin, heparin, or P2Y12 inhibitors clopidogrel (14). The IMPACT II trial, a randomized, double-blind, placebo-controlled Phase III study involving 4,010 patients undergoing elective, urgent, or emergency PCI across 84 U.S. centers (November 1993–November 1994), demonstrated that eptifibatide significantly reduced the composite endpoint of death, myocardial infarction, and urgent revascularization (10). Adeoye et al. (15) reported that eptifibatide combined with recombinant tissue-type plasminogen activator (r-tPA) showed promising efficacy in acute ischemic stroke. Berthelsen et al. (16) found that eptifibatide significantly improved Sequential Organ Failure Assessment (SOFA) scores in septic shock. Lê et al. (17) observed that eptifibatide suppressed influenza virus-induced lung injury in mice. Collectively, these data underscore a promising therapeutic rationale for the potential use of eptifibatide in ARDS treatment.

Due to its rapid onset and reversible inhibition, eptifibatide is well-suited for short-term antiplatelet therapy and is widely used as an intravenous antiplatelet agent in ACS interventions. Its main adverse effects include bleeding (e.g., access site hemorrhage, gastrointestinal bleeding), thrombocytopenia (incidence 1%−2%), and hypotension, with dose adjustments required in renal impairment (halved if CrCl < 50 ml/min) to avoid accumulation. In clinical practice, it is often combined with dual antiplatelet therapy and anticoagulants, necessitating careful monitoring of bleeding risks and drug interactions. The FDA Adverse Event Reporting System (FAERS), the world's largest pharmacovigilance database, provides critical post-marketing drug safety data. This study aims to systematically analyze eptifibatide-related adverse events (AEs) in FAERS using data mining techniques and disproportionality measures to identify potential safety signals, thereby offering evidence-based guidance for clinical use.

## 2 Materials and methods

### 2.1 Data source

This study was conducted based on raw data from the FAERS. This database adopts a voluntary reporting mechanism, primarily receiving adverse drug event reports submitted by healthcare professionals, pharmacists, and patients. The research included all reports from the first quarter of 2004 to the fourth quarter of 2024 that listed eptifibatide as the primary suspected drug, with the data format being original ASCII data packages.

### 2.2 Data management and study design

**Figure 1:**
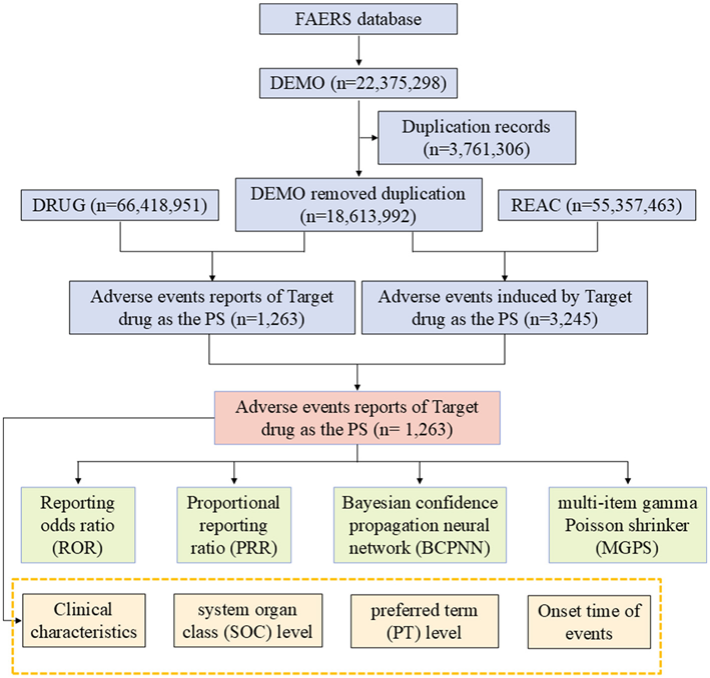
Flowchart demonstrating the AE analysis process for Eptifibatide using the FAERS database. AE, adverse event; DEMO, demographic data; DRUG, drug; FAERSFDA, Adverse Event Reporting System; REAC, reaction data; ROR, reporting odds ratio; PRR, proportional reporting ratio; BCPNN, Bayesian confidence propagation neural network; MGPS, multi-item gamma Poisson shrinker; SOC, system organ class; PS, primary suspect; PT, preferred term.

The data management process included the following key steps: (1) implementation of deduplication processing, strictly following FDA-recommended standard operating procedures. Specifically, reports were first sorted by the unique case identifier (CASEID), retaining the most recent report record based on the FDA receipt date (FDA_DT); for reports with identical CASEID and FDA_DT, the record with the larger PRIMARYID was retained. (2) Starting from the first quarter of 2019, each quarterly data update package included a dedicated deletion report list, which we used to further verify and eliminate duplicate data. (3) The latest released MedDRA dictionary (version 27.1) was used to standardize all adverse event terms, including standardized Preferred Terms (PT) and System Organ Class (SOC) information. The complete study design is shown in the flowchart in Figure 1.

### 2.3 Statistical analysis methods

This study employed a multi-level statistical analysis approach: (1) at the basic level, descriptive statistical analysis was conducted to comprehensively summarize the basic characteristics of adverse event reports. (2) Four advanced disproportionality analysis methods (ROR, PRR, BCPNN, and MGPS) were used for signal mining, with the PRR method's judgment threshold referencing the standards set by the UK MHRA. (3) A Weibull distribution model was established to deeply analyze the temporal distribution characteristics of AEs. All statistical analyses were completed using the professional statistical software SAS 9.4, with technical details of the specific analysis methods and related calculation formulas detailed in Supplementary Tables S1, S2.

## 3 Results

### 3.1 Clinical characteristics

This study included a total of 1,263 adverse event reports (comprising 3,245 AEs) listing eptifibatide as the primary suspected drug. Among these, 24.07% were female patients, 55.11% were male patients, and 20.82% had unspecified gender. The ≥65 age group accounted for the highest proportion (31.35%), followed by the 45–64 age group (27.16%), with 36.82% having unspecified age. Most reports were submitted by physicians (42.04%), pharmacists (27.16%), other healthcare professionals (18.21%), and consumers (9.11%). Severe and non-severe cases accounted for 84.24 and 15.76%, respectively. AEs were mainly concentrated in the period immediately after the drug's market launch from 2004 to 2009, with the annual occurrence of AEs showing a generally declining trend. 72.84% of reports came from the United States. Adverse reactions mostly resulted in patient hospitalization (41.33%), death (17.97%), or life-threatening conditions (15.28%). Details are shown in Table 1.

**Table 1:** Clinical characteristics of AE reports related to Eptifibatide from the FAERS database (Q1 2004-Q4 2024).

**Characteristics**	**Number of cases**	**Proportion of cases (%)**
Number of AE reports	1263	
Number of AEs induced by Eptifibatide	3245	
**Sex**		
Male	696	55.11
Female	304	24.07
Not specified	263	20.82
**Age**		
Median (interquartile range)	64 (56, 73)	
< 18	8	0.63
18–44	51	4.04
45–64	343	27.16
≥65	396	31.35
Not specified	465	36.82
**Reporter**		
Consumer	115	9.11
Lawyer	3	0.24
Other health professional	230	18.21
Pharmacist	343	27.16
Physician	531	42.04
Not specified	41	3.25
**Severity**		
Severe	1,064	84.24
Non-severe	199	15.76
**Reporting year**		
2004	185	14.65
2005	159	12.59
2006	102	8.08
2007	55	4.35
2008	71	5.62
2009	96	7.60
2010	36	2.85
2011	63	4.99
2012	39	3.09
2013	99	7.84
2014	45	3.56
2015	45	3.56
2016	31	2.45
2017	44	3.48
2018	16	1.27
2019	48	3.80
2020	39	3.09
2021	45	3.56
2022	19	1.50
2023	15	1.19
2024	11	0.87
**Top 5 reporting countries**		
United States of America	394 ()	31.20
Russia	6 ()	0.48
Canada	6 (0.48)	0.48
France	4 (0.)	0.32
Qatar	4 (0.32)	0.32

### 3.2 Adverse event distribution at the SOC level

**Figure 2:**
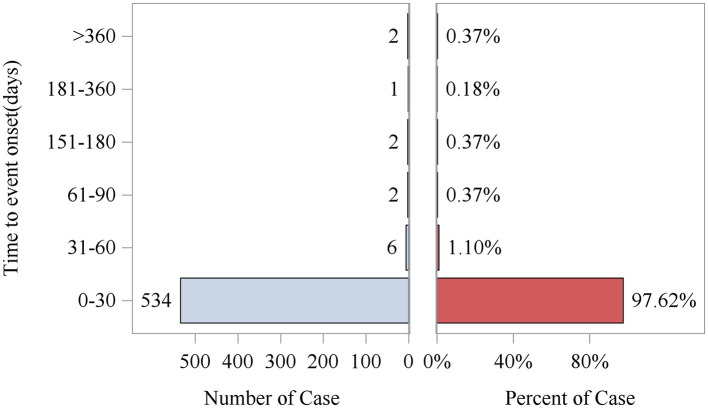
Time to event report distribution of AE reports. AE, adverse event.

Eptifibatide-related AEs involved 16 out of 27 SOC classifications. As shown in Table 2, significantly identified categories included but were not limited to: various injuries, poisoning, and procedural complications; blood and lymphatic system disorders; general disorders and administration site conditions; cardiac disorders; various investigations; vascular disorders; gastrointestinal disorders; nervous system disorders; respiratory, thoracic, and mediastinal disorders; skin and subcutaneous tissue disorders; renal and urinary disorders. Table 2 and Figure 2 display the signal strength at the SOC level.

**Table 2:** Signal strength of Regadenoson-related AEs at the System Organ Class (SOC) level in the FAERS database.

**System Organ Class (SOC)**	**Case reports**	**ROR (95% CI)**	**PRR (95% CI)**	**PRR (χ^2^)**	**IC (IC025)**	**EBGM (EBGM05)**
Nervous system disorders	2,110	2.65 (2.53, 2.78)	2.33 (2.24, 2.42)	1,744.07	1.22 (1.15)	2.33 (2.22)
General disorders and administration site conditions	1,708	0.90 (0.86, 0.95)	0.92 (0.88, 0.96)	14.94	−0.12 (−0.20)	0.92 (0.87)
Gastrointestinal disorders	1,359	1.57 (1.48, 1.66)	1.50 (1.43, 1.57)	246.15	0.58 (0.50)	1.50 (1.42)
Cardiac disorders	1,336	5.28 (4.99, 5.60)	4.75 (4.52, 4.99)	4,056.08	2.25 (2.16)	4.74 (4.48)
Investigations	826	1.28 (1.19, 1.38)	1.26 (1.18, 1.35)	47.15	0.33 (0.23)	1.26 (1.17)
Respiratory, thoracic and mediastinal disorders	693	1.41 (1.30, 1.52)	1.38 (1.29, 1.48)	76.60	0.47 (0.35)	1.38 (1.28)
Vascular disorders	534	2.42 (2.21, 2.64)	2.34 (2.16, 2.55)	420.65	1.23 (1.10)	2.34 (2.15)
Musculoskeletal and connective tissue disorders	526	0.95 (0.87, 1.04)	0.95 (0.88, 1.04)	1.31	−0.07 (−0.20)	0.95 (0.87)
Injury, poisoning and procedural complications	452	0.38 (0.35, 0.42)	0.41 (0.37, 0.45)	427.33	−1.29 (−1.42)	0.41 (0.37)
Skin and subcutaneous tissue disorders	309	0.52 (0.47, 0.59)	0.54 (0.48, 0.60)	128.95	−0.89 (−1.06)	0.54 (0.48)
Psychiatric disorders	211	0.34 (0.29, 0.39)	0.35 (0.31, 0.40)	269.95	−1.51 (−1.71)	0.35 (0.31)
Product issues	157	0.91 (0.78, 1.07)	0.91 (0.78, 1.07)	1.32	−0.13 (−0.36)	0.91 (0.78)
Eye disorders	129	0.60 (0.51, 0.72)	0.61 (0.51, 0.72)	33.31	−0.72 (−0.97)	0.61 (0.51)
Immune system disorders	113	0.96 (0.80, 1.16)	0.96 (0.80, 1.16)	0.15	−0.05 (−0.32)	0.96 (0.80)
Renal and urinary disorders	52	0.25 (0.19, 0.33)	0.26 (0.19, 0.34)	115.00	−1.97 (−2.35)	0.26 (0.19)
Infections and infestations	30	0.05 (0.04, 0.07)	0.05 (0.04, 0.08)	527.41	−4.22 (−4.69)	0.05 (0.04)
Metabolism and nutrition disorders	24	0.10 (0.07, 0.15)	0.10 (0.07, 0.15)	190.11	−3.27 (−3.79)	0.10 (0.07)
Surgical and medical procedures	21	0.14 (0.09, 0.22)	0.15 (0.10, 0.22)	105.75	−2.77 (−3.33)	0.15 (0.10)
Social circumstances	18	0.36 (0.23, 0.58)	0.36 (0.23, 0.58)	20.18	−1.46 (−2.07)	0.36 (0.23)
Reproductive system and breast disorders	15	0.16 (0.09, 0.26)	0.16 (0.10, 0.26)	68.16	−2.67 (−3.31)	0.16 (0.09)
Ear and labyrinth disorders	14	0.30 (0.18, 0.51)	0.30 (0.18, 0.51)	22.67	−1.73 (−2.40)	0.30 (0.18)
Blood and lymphatic system disorders	9	0.05 (0.03, 0.09)	0.05 (0.03, 0.10)	165.00	−4.32 (−5.09)	0.05 (0.03)
Hepatobiliary disorders	6	0.06 (0.03, 0.14)	0.06 (0.03, 0.14)	86.99	−4.03 (−4.91)	0.06 (0.03)
Congenital, familial and genetic disorders	4	0.12 (0.05, 0.33)	0.12 (0.05, 0.33)	24.80	−3.01 (−4.02)	0.12 (0.05)
Neoplasms benign, malignant and unspecified (incl cysts and polyps)	4	0.01 (0.01, 0.04)	0.01 (0.01, 0.04)	280.62	−6.13 (−7.11)	0.01 (0.01)
Endocrine disorders	3	0.11 (0.04, 0.34)	0.11 (0.04, 0.34)	21.44	−3.17 (−4.25)	0.11 (0.04)

Ranked by case reports.

### 3.3 Adverse event distribution at the PT level

Eptifibatide-related AEs were sorted by frequency and evaluated for signals. Among the top 50 most common AEs, known common side effects included bleeding (puncture site hemorrhage, gastrointestinal hemorrhage, hematuria, intracranial hemorrhage, gingival bleeding, and epistaxis), thrombocytopenia, and hypotension. Severe adverse reactions (rare but possible) included major bleeding (intracranial hemorrhage, retroperitoneal hematoma) and acute severe thrombocytopenia. Additionally, potential AEs not listed in the drug label were identified, such as hemorrhagic pancreatitis, coronary artery reocclusion, vascular stent thrombosis, anisocoria, brain herniation, cardiac arrest, and other newly emerged AEs. Table 3 shows details of the top 50 AEs at the PT level. All AEs meeting the positive signal criteria are listed in Supplementary Table S3.

**Table 3:** Top 50 most frequent AEs for Regadenoson at the preferred term (PT) level.

**Preferred term (PT)**	**Case reports**	**ROR (95% CI)**	**PRR (95% CI)**	**PRR (χ^2^)**	**IC (IC025)**	**EBGM (EBGM05)**
Nausea	483	3.68	3.55	897.56	1.83	3.55
Dyspnea	375	3.93	3.83	789.54	1.94	3.82
Injection site extravasation	355	162.23	156.87	53,349.2	7.25	152.21
Vomiting	344	4.41	4.3	876.18	2.1	4.29
Headache	291	2.72	2.67	307.35	1.42	2.67
Hypotension	289	8.52	8.31	1,862.23	3.05	8.3
Cardiac arrest	284	19.93	19.43	4,951.44	4.27	19.36
Seizure	282	9.59	9.36	2,109.03	3.22	9.35
Dizziness	246	2.89	2.85	296.6	1.51	2.84
Chest pain	205	6.35	6.25	905.49	2.64	6.24
Blood pressure decreased	201	17.75	17.43	3,106.12	4.12	17.38
Bradycardia	192	20.69	20.33	3,518.35	4.34	20.26
Loss of consciousness	176	7.97	7.86	1,053.63	2.97	7.85
Tremor	166	5.74	5.66	638.21	2.5	5.66
Unresponsive to stimuli	149	33.79	33.34	4,645.13	5.05	33.13
Incorrect product administration duration	131	14.97	14.8	1,681.96	3.88	14.76
Pain in extremity	131	2.53	2.51	119.33	1.33	2.51
Heart rate increased	116	6.82	6.76	568.99	2.75	6.75
Heart rate decreased	100	16.19	16.05	1,407.55	4	16
Syncope	99	5.62	5.58	372.16	2.48	5.57
Chest discomfort	96	5.57	5.53	356.72	2.47	5.53
Hyperhidrosis	82	3.62	3.6	154.35	1.85	3.6
Blood pressure increased	78	2.93	2.92	98.6	1.54	2.92
Atrioventricular block	75	56.4	56.01	4,008.67	5.79	55.41
Atrioventricular block complete	74	64.05	63.61	4,504.58	5.97	62.84
Presyncope	66	16.07	15.97	923.9	3.99	15.93
Flushing	63	3.46	3.45	109.44	1.78	3.44
Infusion site extravasation	58	54.33	54.04	2,988.11	5.74	53.49
Atrial fibrillation	50	2.95	2.94	64.02	1.55	2.94
Extravasation	46	63.85	63.58	2,798.75	5.97	62.81
Atrioventricular block second degree	44	82.72	82.39	3,481.41	6.34	81.09
Wheezing	42	4.43	4.42	111.17	2.14	4.42
Sinus arrest	40	153.34	152.77	5,855.58	7.21	148.35
Electrocardiogram ST segment depression	36	91.88	91.57	3,168.08	6.49	89.97
Acute myocardial infarction	36	6.72	6.7	174.37	2.74	6.69
Cardio-respiratory arrest	36	4.75	4.74	106.18	2.24	4.74
Respiratory arrest	35	6.88	6.86	175.19	2.78	6.86
Retching	32	8.79	8.76	219.78	3.13	8.75
Pallor	31	6.42	6.41	141.36	2.68	6.4
Aphasia	31	5.73	5.72	120.59	2.51	5.71
Product administration error	31	3.39	3.38	51.95	1.76	3.38
Bronchospasm	28	11.1	11.08	256.22	3.47	11.06
Ventricular extrasystoles	27	14.45	14.42	336.34	3.85	14.38
Electrocardiogram ST segment elevation	26	43.84	43.73	1,076.46	5.44	43.37
Electrocardiogram abnormal	25	17.67	17.63	390.97	4.14	17.58
Throat tightness	25	5.36	5.35	88.31	2.42	5.34
Infusion site pain	24	11.8	11.77	236.09	3.55	11.75
Nodal rhythm	23	87.83	87.64	1,936.83	6.43	86.18
Ventricular tachycardia	23	7.95	7.94	139.33	2.99	7.93
Supraventricular tachycardia	22	13.02	12.99	242.98	3.7	12.96

### 3.4 Timing of adverse event occurrence

**Figure 3:**
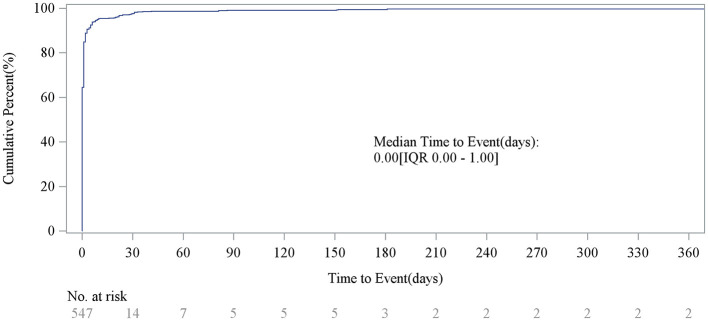
Cumulative incidence of AEs. AE, adverse event.

After excluding reports with false or missing adverse event timing data, 547 eptifibatide-related AEs provided occurrence time data. Almost all AEs occurred immediately after administration (*n* = 534, 97.62%). The median time to onset was 0 days [interquartile range (IQR) 0.00–0.00 days]. Rare cases occurred 2 months after administration (*n* = 6, 1.10%), 3 months after (*n* = 2, 0.37%), and between 5 and 12 months after (*n* = 5, 0.81%). The temporal distribution is shown in Figure 1, and the cumulative incidence curve is shown in Figure 3.

## 4 Discussion

This investigation comprehensively evaluated AEs associated with eptifibatide since its market launch in 2004. Through analysis of the FAERS database, the study confirmed common AEs listed in the drug label, including bleeding (puncture site hemorrhage, gastrointestinal hemorrhage, hematuria, intracranial hemorrhage, gingival bleeding, and epistaxis), thrombocytopenia, and hypotension, as well as severe bleeding (intracranial hemorrhage, retroperitoneal hematoma) and acute severe thrombocytopenia. Additionally, potential AEs not listed in the label were identified, such as hemorrhagic pancreatitis, coronary artery reocclusion, vascular stent thrombosis, anisocoria, brain herniation, and cardiac arrest. These results highlight the importance of enhanced drug monitoring, particularly during the initial administration of eptifibatide, to effectively manage potential side effects.

Our findings suggest that eptifibatide use may be associated with hemorrhagic pancreatitis, a severe pancreatic pathology involving tissue damage, inflammation, and necrosis, consistent with the clinical spectrum of acute or chronic pancreatitis (18). Hemorrhagic pancreatitis is a severe form of pancreatitis, possibly related to pancreatic ischemia and hypoxia, activation of pancreatic enzymes, inflammatory mediators, and increased intrapancreatic tissue pressure (19). These factors lead to vascular damage and bleeding, with severe cases resulting in shock and multiple organ failure. Studies have found that known adverse reactions of eptifibatide include bleeding (20). A plausible explanation is that eptifibatide administration may cause pancreatic tissue ischemia and hypoxia, leading to pancreatic enzyme activation and inflammatory responses that promote the development of hemorrhagic pancreatitis. Alternatively, bleeding may induce inflammatory responses and pancreatic enzyme activation, exacerbating pancreatitis. The relationship between eptifibatide and hemorrhagic pancreatitis requires further research to confirm. Therefore, awareness of this potential adverse effect underscores the importance of monitoring pancreatic function during eptifibatide therapy in ARDS to enable timely intervention and prevent hemorrhagic pancreatitis. Coronary artery reocclusion represents another potential adverse reaction, referring to the recurrence of vascular occlusion detected during follow-up angiography after successful thrombolysis of occluded arteries in AMI (21). This complication predominantly occurs within 1 week post-thrombolysis with an incidence of approximately 5%−15%, increasing to about 35% at 6 months after thrombolytic therapy, and is clinically associated with post-thrombolytic angina, arrhythmias, cardiac function deterioration, and mortality (22). The underlying mechanism involves thrombolytic therapy dissolving clots at ruptured plaques while leaving the original plaques intact, combined with residual coronary stenosis, highly thrombogenic residual thrombi, and re-exposure of ruptured surfaces, ultimately leading to thrombus reformation (23). These pathophysiological processes correlate with eptifibatide resistance, plaque rupture, and persistent coagulation activation of residual mural thrombi. Consequently, eptifibatide administration may result in suboptimal antiplatelet effects, thereby elevating patients' risk of coronary reocclusion. Therefore, clinicians should exercise particular caution regarding this potential adverse reaction when prescribing eptifibatide.

Stent thrombosis has been identified as a potential adverse effect of eptifibatide, manifesting as acute or subacute in-stent thrombosis following emergency percutaneous intervention and antiplatelet therapy in AMI patients, representing a serious post-procedural complication (24). The etiology is multifactorial, potentially involving inappropriate stent sizing, vascular dissection formation, incomplete plaque coverage, antiplatelet drug resistance, and drug interactions (25). Some patients exhibit inadequate suppression of platelet aggregation despite long-term standard-dose clopidogrel therapy, a phenomenon termed clopidogrel hypo-responsiveness or resistance (26). Eptifibatide may demonstrate analogous drug resistance patterns. The identification of stent thrombosis as a potential eptifibatide-related adverse reaction, distinct from its established post-marketing adverse effects, provides valuable guidance for clinical safety monitoring during eptifibatide therapy.

Our research additionally suggests anisocoria (a pupillary diameter disparity exceeding 1 mm between eyes) as a potential risk associated with eptifibatide (27). While physiological causes include hereditary factors, aging, and light adaptation, pathological etiologies encompass cerebral hemorrhage, brain tumors, iritis, mydriatic agents (e.g., atropine), and Horner's syndrome (28). Clinicians should remain vigilant for accompanying symptoms such as acute visual deterioration, eyelid dysfunction, sudden dizziness, headache, and nausea (29), which correlate with eptifibatide's known adverse effects of severe bleeding and intracranial hemorrhage. Intracranial hemorrhage-induced anisocoria signifies critical illness, often indicating direct brainstem or thalamic hemorrhage affecting pupillary regulatory centers, oculomotor nerve compression from elevated intracranial pressure, or cerebral herniation compressing cranial nerves and brainstem (30). Vigilant monitoring for pupillary changes, particularly anisocoria as a potential indicator of intracranial hemorrhage, is critical during eptifibatide infusion, and patients should be counseled accordingly about this risk. Our study identified brain herniation as a potential adverse reaction to eptifibatide. The cranial cavity is a fixed-volume enclosed space containing brain tissue, cerebrospinal fluid, and blood, where any increase in intracranial components can elevate local or generalized pressure, causing displacement of brain tissue to lower-pressure areas and forming life-threatening herniation, with the most common types being transtentorial, subfalcine, and tonsillar herniation (31). Common etiologies include intracranial hematomas, tumors, abscesses, parasitic infections, and congenital malformations, which correlate with eptifibatide's known adverse effect of intracranial hemorrhage (32). A plausible explanation is that eptifibatide administration may induce intracranial hemorrhage, with blood accumulation increasing local pressure that compresses and damages brain tissue, promoting displacement and herniation (33). While further research is needed to confirm the relationship between eptifibatide and brain herniation, this finding provides clinicians with a rationale for monitoring intracranial pressure and preventing herniation in patients receiving eptifibatide therapy.

Cardiac arrest, also known as asystole, was identified as another potential adverse reaction, representing complete loss of cardiac electrical activity and a primary cause of sudden cardiac arrest (34). Etiologies can be cardiac (e.g., coronary artery disease, cardiomyopathy, myocarditis) or non-cardiac (e.g., severe cerebral hemorrhage, pulmonary embolism, acute poisoning, hemorrhagic shock, anaphylaxis, electrocution, hyperkalemia, severe acidosis), corresponding with eptifibatide's known effects, including severe bleeding, allergic reactions, and intracranial hemorrhage (35). The pathways to cardiac arrest in this setting include exsanguination leading to hemorrhagic shock, anaphylaxis culminating in distributive and cardiogenic shock, and intracranial hemorrhage causing cerebral herniation via elevated intracranial pressure. Although more clinical studies are needed to investigate this association, our findings suggest cardiac arrest represents a potential eptifibatide-related adverse event warranting close cardiac monitoring and prompt intervention during treatment.

Through disproportionality analysis of FAERS data, we characterized the cumulative incidence and temporal patterns of adverse reactions primarily attributed to eptifibatide. These results emphasize the importance of early detection and management of treatment-related complications following eptifibatide administration for ARDS and other conditions, providing valuable evidence for adverse reaction prevention and mitigation to improve clinical outcomes.

This study has limitations inherent to its data source. The FAERS database, which relies on spontaneous reports, is susceptible to under-reporting, reporting biases, and variable data quality, which may affect the completeness and accuracy of our analysis. We implemented rigorous quality control measures, including correction of obvious data entry errors and duplicate report removal, to enhance reliability. Second, the exclusively U.S.-derived data may limit generalizability to other regions like Asia, Africa, and Latin America. Finally, while FAERS effectively identifies potential adverse reaction risks, its utility for epidemiological analysis remains limited.

## 5 Conclusions

This study utilized FAERS data to conduct disproportionality analysis of eptifibatide-related adverse events, focusing on all reports from the first quarter of 2004 to the fourth quarter of 2024. The analysis not only confirmed known adverse reactions of eptifibatide including bleeding (puncture site hemorrhage, gastrointestinal hemorrhage, hematuria, intracranial hemorrhage, gingival bleeding, and epistaxis), thrombocytopenia, and hypotension, as well as severe bleeding (intracranial hemorrhage, retroperitoneal hematoma) and acute severe thrombocytopenia, but also identified several potential adverse reactions such as hemorrhagic pancreatitis, coronary artery reocclusion, vascular stent thrombosis, anisocoria, brain herniation, and cardiac arrest. These findings underscore the necessity for early monitoring of adverse reactions in patients to ensure the safe application of eptifibatide in treating ARDS and other conditions.

## Data Availability

The original contributions presented in the study are included in the article/Supplementary material, further inquiries can be directed to the corresponding author.
